# Neuropeptide Substance P Improves Osteoblastic and Angiogenic Differentiation Capacity of Bone Marrow Stem Cells *In Vitro*

**DOI:** 10.1155/2014/596023

**Published:** 2014-06-23

**Authors:** Su Fu, Gang Mei, Zhao Wang, Zhen-Lv Zou, Song Liu, Guo-Xian Pei, Long Bi, Dan Jin

**Affiliations:** ^1^Department of Orthopedics and Traumatology, Nanfang Hospital, Southern Medical University, 1838 North Guangzhou Avenue, Guangzhou, Guangdong 510515, China; ^2^School of Engineering and Materials Science, Queen Mary University of London, Mile End Road, London E1 4NS, UK; ^3^Department of Orthopedic Surgery, Xijing Hospital, The Fourth Military Medical University, Xi'an 710032, China

## Abstract

Our previous work showed that implanting a sensory nerve or vascular bundle when constructing vascularized and neurotized bone could promote bone osteogenesis in tissue engineering. This phenomenon could be explained by the regulatory function of neuropeptides. Neuropeptide substance P (SP) has been demonstrated to contribute to bone growth by stimulating the proliferation and differentiation of bone marrow stem cells (BMSCs). However, there have been no prior studies on the association between Wnt signaling and the mechanism of SP in the context of BMSC differentiation. Our results have shown that SP could enhance the differentiation of BMSCs by activating gene and protein expression via the Wnt pathway and by translocating *β*-catenin, which can be inhibited by Wnt signaling blocker treatment or by the NK-1 antagonist. SP could also increase the growth factor level of bone morphogenetic protein-2 (BMP-2). Additionally, SP could enhance the migration ability of BMSCs, and the promotion of vascular endothelial growth factor (VEGF) expression by SP has been studied. In conclusion, SP could induce osteoblastic differentiation via the Wnt pathway and promote the angiogenic ability of BMSCs. These results indicate that a vascularized and neurotized tissue-engineered construct could be feasible for use in bone tissue engineering strategies.

## 1. Introduction

Bone grafting, typically autologous bone grafting, is commonly utilized to repair skeletal defects in the reconstruction of bone integrity [1]. Although it is regarded as the gold standard [2, 3], autologous bone grafting can cause complications such as morbidity and infection; moreover, only limited amounts of autograft material are available and the harvesting process involves additional trauma [1, 4], producing an unsatisfactory outcome [5]. Bone tissue engineering has been successfully developed and has successfully provided bone substitute in reconstructive orthopedics [5]. However, improving bone tissue-engineered growth and angiogenesis remains a challenge [6]. Thus, there is an ongoing need for information on the regulatory mechanisms of osteoblastic differentiation and angiogenesis.

Previous studies have shown that the implantation of a sensory nerve or vascular bundle when constructing engineered vascularized and neurotized bone tissue could promote osteogenesis [7, 8]. Several studies have since investigated the effect and underlying mechanism of sensory nerves. The neurotized bone tissues showed a higher degree of osteoblastic differentiation and angiogenesis than in vivo engineered bone tissue grafts alone, indicating that nerve fibers are directly involved in bone growth. The effect on the neurotized bone was similar to the effect of neuropeptide action on vascularized bone implantation [7]. The early expression of neuropeptide receptors was also significantly improved by implanting vascular bundles. Similarly, the neuropeptides (such as calcitonin gene-related peptide (CGRP) and substance P (SP)) released by the sensory nerve have been found to contribute to bone formation by acting on bone marrow stem cells (BMSCs) in vitro [9, 10]. These data indicate that neurotization treatment could stimulate bone generation via neuropeptides, which play important roles in bone tissue formation. However, the mechanism of this stimulation remains unknown. The functioning of engineered bone tissue has depended on the osteogenic differentiation and vascularization of the seeding cells (BMSCs) [11]. Further data are needed to understand how neuropeptide SP affects the osteoblastic differentiation and angiogenesis of BMSCs.

The osteoblastic differentiation of BMSCs in engineered bone tissue involves several cellular processes including osteoblastic gene expression. Bone morphogenetic protein-2 (BMP-2) has been found to be the major regulating factor in inducing bone formation in engineered bone tissue [12–14] and is preferred for the growth factor delivery system [15]. Additionally, the expression of osteoblastic genes and proteins is vital to bone generation [16], and Wnt signaling is an important regulatory factor in bone metabolism [17–19]. SP has been confirmed to exert a promoting effect on the proliferation and differentiation of BMSCs [20–24]. However, the effect of SP on BMP-2 expression is currently unclear. Additionally, the relationship between SP and canonical Wnt signaling in the context of osteogenic processes has not been clarified. The effects of SP on the Wnt pathway and the expression of BMP-2 were investigated in this study.

Angiogenesis in engineered bone tissue is closely related to osteogenesis and always precedes bone formation [25, 26]. Vascular endothelial growth factor (VEGF) plays a fundamental role in promoting angiogenesis [27] and is also the first choice for use in growth factor delivery systems. Stable vessels in vivo were achieved through the continuous release of VEGF from the scaffold [28]. VEGF functions as a potent angiogenic peptide, but the migration of BMSCs is also required for the formation of new vessels [29, 30]. The migration of cells to the site of injury is the foundation of the angiogenesis process. The VEGF expression and migration ability were both viewed as the critical promoting factor in angiogenesis process and the representation of angiogenic capacity of BMSCs. However, there have been no prior studies on the effects of SP on the migration and VEGF expression of BMSCs. Therefore, the angiogenic capacity of BMSCs (including migration ability and VEGF expression, influenced by SP) was also evaluated in this study.

## 2. Materials and Methods

### 2.1. Preparation of BMSCs

Rat BMSCs were isolated from the bone marrow of femoral and tibial medullary cavities of 80–100 g rats and were flushed using ice-cold L-Dulbecco's Modified Eagle's Medium (L-DMEM) (Gibco, USA) supplemented with 10% fetal bovine serum (FBS, Gibco, Australia). The suspension of flushed marrow cells was passed repeatedly through a 22-gauge needle and filtered through a 100 *μ*m cell strainer before culturing. The marrow cells were grown in 25 cm^2^ tissue culture flasks with an appropriate number of 1 × 10^6^–1 × 10^7^ cells/mL. The cultures were incubated at 37°C in a humidified incubator containing 5% CO_2_. The medium was L-DMEM supplemented with 10% FBS, 100 IU/mL penicillin, 100 *μ*g/mL streptomycin, and 1 *μ*g/mL amphotericin and was changed every 2 days. When the cell confluence reached 80%, the BMSCs were passaged with 0.02% trypsin (Gibco, USA) and transferred to new culture flasks at a ratio of 1 : 2. The cells were cultured in 24-well culture plates at 37°C in a humidified atmosphere containing 5% CO_2_.

### 2.2. BMSCs Identification and Differentiation

**Figure 1:**
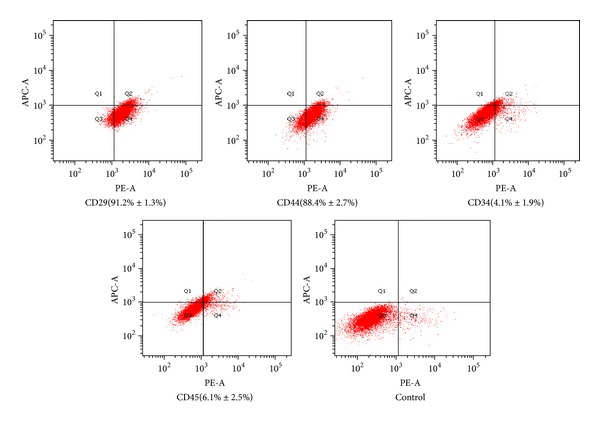
Identify of BMSCs. The BMSCs were identified with CD29 (91.2% ± 1.3%), CD44 (88.4% ± 2.7%), CD34 (4.1% ± 1.9%), and CD45 (6.1% ± 2.5%).

After three passages, the culture medium was changed to a differentiation medium (L-DMEM containing 10 mM *β*-glycerophosphate, 100 nm dexamethasone, and 50 *μ*g/mL ascorbic acid) to induce osteogenic differentiation [31]. The expression of CD29, CD34, CD44 and CD45 were analyzed via flow cytometry for detecting the purity of BMSCs (Figure 1) [32, 33]. Approximately 5.0 × 10^4^ purified BMSCs were stained with 20 mL phycoerythrin (PE)-conjugated anti-CD29, CD34, CD44, and CD45 for 30 min at 4°C. PE-labeled antibody to rat IgG1 was used as a control. The cells were washed twice with ice-cold phosphate-buffered saline (PBS), fixed in 1.0% paraformaldehyde and analyzed via flow cytometry within 24 h. Fluorescence was analyzed with a Coulter Elite-ESP flow cytometer (Beckman-Coulter Electronics, Hialeah, FL) using Elite software.

### 2.3. Groups

For detection of the effects of SP on the differentiation of BMSCs, the concentration of SP (10^−12^ mol/L) and treatment duration (7 days and 14 days) were based on previous research [24]. To further clarify the relationship between the Wnt pathway and SP during BMSC differentiation, four groups were created: Group A was the control group, and the same amount of PBS was added; in Group B, the culture was incubated with SP (the concentration to be determined by the results of step one); in Group C, the culture was incubated with a mixture of SP and NK1 antagonist (1 *μ*M Sigma CP-96345 USA) [23, 34]; and in Group D, the culture was incubated with a mixture of SP and 0.2 *μ*g/mL DKK1 (0.2 *μ*g/mL Peprotech recombinant Human DKK-1 (Dickkopf-related protein-1), USA) [35]. To identify the first changes in the expression of BMP-2 and VEGF, observations were made on 1 day, 3 days, 5 days, and 7 days; these intervals were also employed to determine the effects of SP on the migration of the BMSCs. The concentration of SP (10^−8^ mol/L) was chosen for the possible maximum effect [20].

### 2.4. Immunocytochemical Staining

The cells positive for nuclear BMP-2, VEGF, and *β*-catenin were detected via immunocytochemical staining. After treatment, the BMSCs were seeded on the coverslip and were allowed to attach overnight. After being fixed in 4% paraformaldehyde in PBS for 15 min at room temperature and then permeabilized in 0.25% Triton X-100 in PBS for 15 min, the cells were incubated in 1% BSA in PBST for 30 min to avoid nonspecific binding of the antibody or in goat serum for 10 min, washed three times in PBS and incubated overnight with primary anti-*β*-catenin antibody (Santa Cruz Biotechnology, Inc.). The cells were then diluted 1 : 100 in PBST or in anti-BMP-2 antibody anti-VEGF antibody (Abcam) diluted 1 : 150 in PBST. After three washes, the cells were incubated for 1 h with FITC-linked secondary antibodies diluted in 1 : 100 USCN. The cells were washed three times in PBS and then with DAPI to identify nuclei and detect the translocation of *β*-catenin. Alternatively, after the secondary antibodies had incubated, the BMSCs were stained with DAB and counterstained with hematoxylin, dehydrated in graded methanol, cleared in xylene, and finally mounted. The slides were examined using fluorescent microscopy at ×40 magnification, and the images were acquired using the Image Manager software.

### 2.5. Quantitative Polymerase Chain Reaction (qPCR)

To validate the gene expression of the BMSCs in all groups, the total RNA was isolated from the cell lines, and cDNA synthesis was performed using TRIzol and Oligo-dT (Invitrogen, USA). The qPCR assay was performed using SYBR Green assays (Applied Biosystems, USA). The amplification conditions were as follows: 95°C for 3 min followed by 40 cycles alternating between 95°C for 15 s and 60°C for 30 s. Thermal cycling and fluorescence detection were performed using the StepOnePlus Real-Time PCR System (Applied Biosystems, USA). The alkaline phosphatase mRNA, collagen type I mRNA, osteocalcin mRNA, Runx2 mRNA, C-myc mRNA, Lef1, Tcf7, and *β*-catenin mRNA expression levels were compared with the GAPDH expression levels using the ΔCt method. All primers for qRT-PCR were designed using the Primer Express software (ABI). The primer sequences used in this study are listed in Table 1. The reported data represent the mean expression from 3 experiments.

### 2.6. Western Blot Analysis

The cells were treated with the lysis buffer (Cell Signaling Technology), and the protein extracts were dissolved in a sample buffer containing 50 mM Tris-HCl, 2% SDS, 10% glycerol, and 100 mM dithiothreitol (pH = 6.80). Proteins were separated using SDS-PAGE in 10% polyacrylamide gel and transferred to a nitrocellulose membrane. Blots were performed with anti-BMP-2 antibody, anti-VEGF antibody, anti-ALP antibody (Abcam), anti-*β*-catenin antibodies, anti-GSK-3*β* antibodies, and anti-C-myc antibodies (Santa Cruz Biotechnology, CA). The bands were captured and documented using a CCD system (Image Station 2000 MM, Kodak, Rochester, NY, USA). The blots were stripped and reprobed with anti-actin antibodies to demonstrate equal loading and to enable between-group protein content normalization. Densitometry of the bands was performed using Molecular Imaging Software Version 4.0 (Kodak).

### 2.7. Scratch Recovery Assay

BMSCs at the logarithmic phase of growth were plated into six-well culture dishes at a density of 10^6^ cells. After incubation for 24 h, the cells were synchronized with 2% FBS. A straight line was then introduced to each well via scratching with a sterile 200 *μ*L pipette tip once the cells had reached 80% confluency. The detached cells were removed by gently washing the well three times with 100 mM PBS (pH 7.4) at 37°C. The cells were then allowed to grow for an additional 24 h in culture medium supplemented with 2% FBS and 100 *μ*mol/L temozolomide or DMSO. Cell migration was photographed at ×100 magnification under a microscope (Olympus, Tokyo, Japan) 1 day later, 3 days later, 5 days later, and 7 days later. The rate of cell migration was accounted according to the established method: the area of migration cells (A) and the cell-free area (B) were calculated and the results were represented as A/A+B.

### 2.8. Transwell Migration Assays

The cell migration assay was performed in a 24-well transwell migration chamber (BD Biosciences, San Jose, CA, USA) with polycarbonate filters (diameter 6.525 mm; pore size 8.25 mm). The upper well contained BMSCs cultured in medium 199 supplemented with 0.5% BSA. The lower well contained media with SP as a chemoattractant. After a 72 h migration at 37°C, all cells that had migrated were fixed in 4% paraformaldehyde/PBS and stained with hematoxylin. The number of stained cells was counted in Image-Pro Plus 4.5 (Media CyberNetics, Silver Spring, Maryland, USA). Each counting was repeated with three holes in the middle and surrounded five horizons under the microscope camera, and the results were expressed in percentage. The cells that did not migrate were removed from the upper surface by scraping with a cotton swab.

### 2.9. Statistical Analysis

Statistical analyses were performed using SPSS software (version 13.0). We performed a one-way ANOVA analysis to compare the means. The comparisons between groups were performed using Dunnett's (2-tailed *t*) post hoc test. Significance was declared if *P* < 0.05. The presented error bars show the standard error of the mean (SEM).

## 3. Results

### 3.1. SP Enhanced the Expression of BMP-2 and the Gene and Protein Expression in BMSC Differentiation

**Figure 2:**
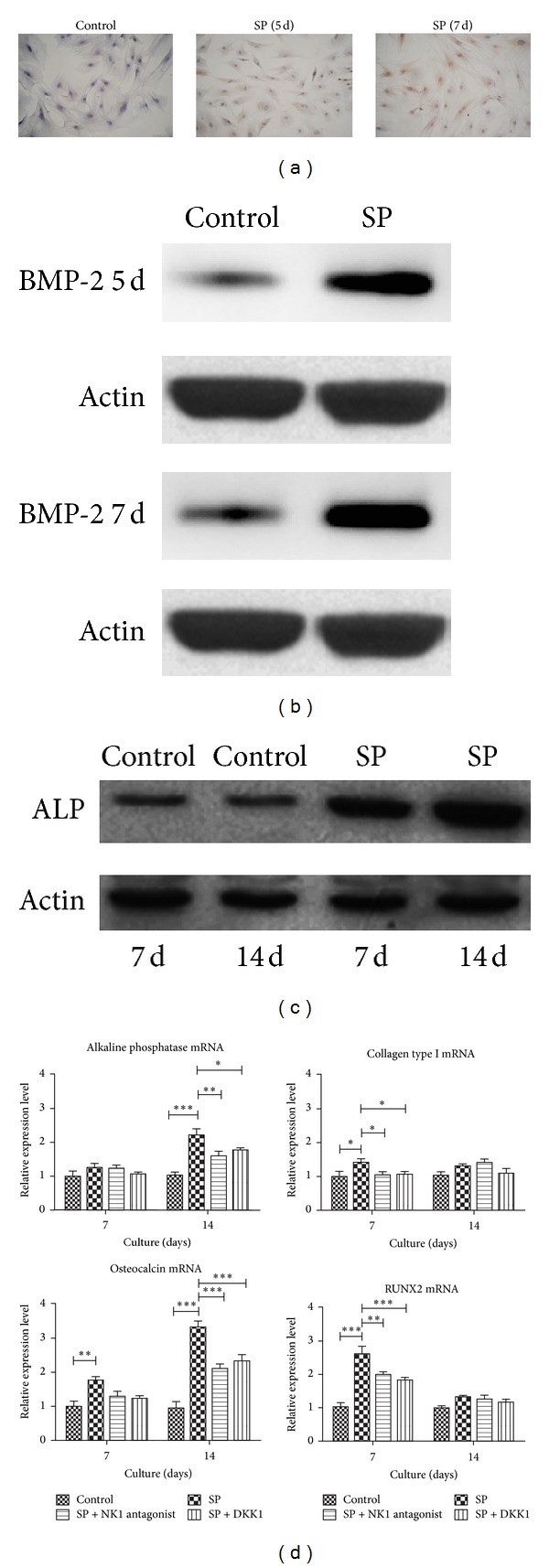
Expression of BMP-2, ALP protein, and genes in BMSC differentiation. SP increased the rates of BMP-2 positive cells (a) and protein BMP-2 expression (b) from day 5 to day 7. SP improved the synthesis of ALP protein (c) and of osteoblastic genes such as ALP, collagen type 1, osteocalcin, and RUNX2 (d). The expression of osteoblastic genes could be inhibited by DKK or NK1 antagonist.

**Figure 3:**
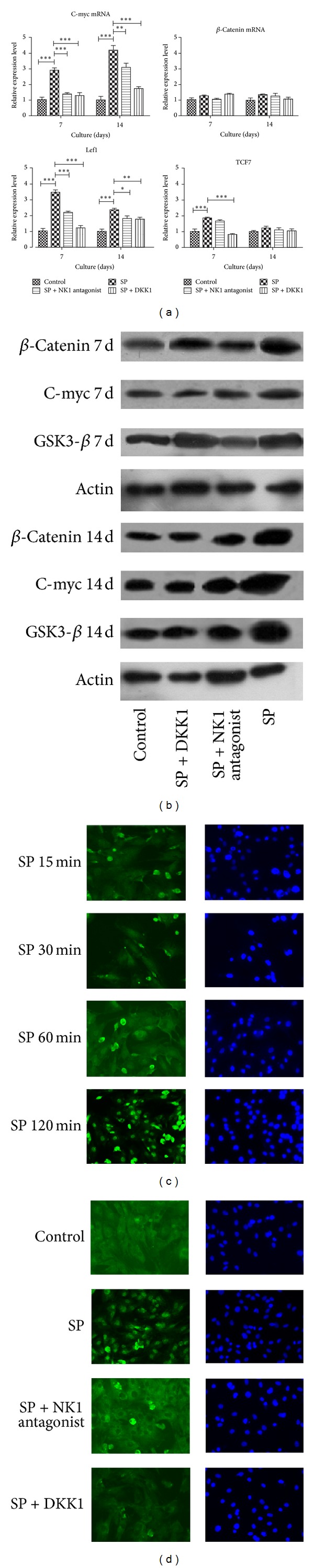
SP activated the expression of Wnt signaling genes and proteins and the translocation of *β*-catenin to the nucleus. SP increased the expressions of the Wnt genes C-myc, Tcf7, and Lef1. When the NK1 receptor was blocked, this effect was significantly decreased compared with the SP group (a). Increases in the Wnt signaling proteins *β*-catenin, C-myc, and p-GSK-3*β* were observed under SP treatment on day 7 and day 14 (b). The nuclear translocation of *β*-catenin occurred after 15 min of SP treatment, (c) which could be blocked by NK1 antagonist or DKK treatment (d).

The immunohistochemistry results in Figure 2(a) show that a positive DAB stain with BMP-2 (the obvious brown area) was observed on the cytoplast and cell nucleus (×400) in SP treatment (10^−8^ mol/L) at 5 days and 7 days; the same result was observed after 7 days in the control group. The rates of the BMP-2-positive cells were increased after SP treatment, and the expression of protein BMP-2 also improved (0.1413 ± 0.0071 compared with 0.0073 ± 0.0025 on 5 days; 0.18 ± 0.004 compared with 0.043 ± 0.003 on 7 days, Figure 2(b)). SP did not affect the rates of the BMP-2-positive cells or BMP-2 expression between day 1 and day 3. The osteoblastic genes (ALP, collagen type 1, osteocalcin, and RUNX2) and protein (ALP) selected to represent the osteoblastic degree were all increased by SP (0.628 ± 0.00225 compared with 0.1027 ± 0.0075 on 7 days; 0.572 ± 0.005 compared with 0.115 ± 0.007 on 14 days, Figures 2(c) and 2(d)). Interestingly, the NK1 receptor antagonist and Wnt pathway antagonist DKK blocked the promotion effect of gene expression (Figure 3(d)). The expression of the Wnt genes (such as C-myc, Tcf7, and Lef1) and Wnt proteins such as *β*-catenin and c-myc was promoted by SP, which could be inhibited via NK1 receptor antagonist treatment. For ALP and osteocalcin mRNA expression, these phenomena were more obvious after 14 days than after 7 days. For collagen type 1 and RUNX2 mRNA expression, the phenomenon was more obvious after 7 days of treatment than after 14 days. The blocking effects were significant but not complete (Figure 2(d)).

### 3.2. SP Induced Osteoblastic Differentiation by Regulating Wnt Signaling in BMSCs

The Wnt pathway inhibitor DKK treatment blocked the osteoblastic gene expression promoting effect of SP (Figure 2(d)), indicating the possible involvement of Wnt signaling. The results in Figure 2 confirmed the increased expression of genes and proteins in the Wnt pathway due to SP treatment; the DKK and NK1 receptor antagonist inhibited the activation of the Wnt pathway. As shown in Figure 3(a), the increased expression of the Wnt genes (such as C-myc, Tcf7, and Lef1) via SP treatment and the blocking effect of the NK1 antagonist and DKK were apparent; however, the mRNA of *β*-catenin expression was not. The western blot results revealed that SP activated the expression of Wnt signaling proteins such as *β*-catenin (1.76 ± 0.31 and 3.12 ± 0.07 fold change compared with control group on 7 days and 14 days), C-myc (1.34 ± 0.50 and 2.29 ± 0.14 fold change compared with control group on 7 days and 14 days), and p-GSK-3*β* (2.45 ± 0.11 and 2.12 ± 0.18 fold change compared with control group on 7 days and 14 days), which was revealed in Figure 3(b). This effect was more obvious after 14 days of treatment than after 7 days. The nuclear translocation of *β*-catenin was the critical event in Wnt signaling activation; it precipitated the obvious activation of nuclear transfer after SP treatment. It was clear after 15 min that this effect was inhibited by the NK1 antagonist and DKK treatment, which proved that SP treatment could activate the Wnt pathway. As shown in Figure 3(d), the nuclear translocation of *β*-catenin did not occur after SP treatment combined with NK1 antagonist treatment; the nucleus was relatively dark compared with the cytoplasm.

### 3.3. SP Stimulated the Migration and VEGF Expression of BMSCs

**Figure 4:**
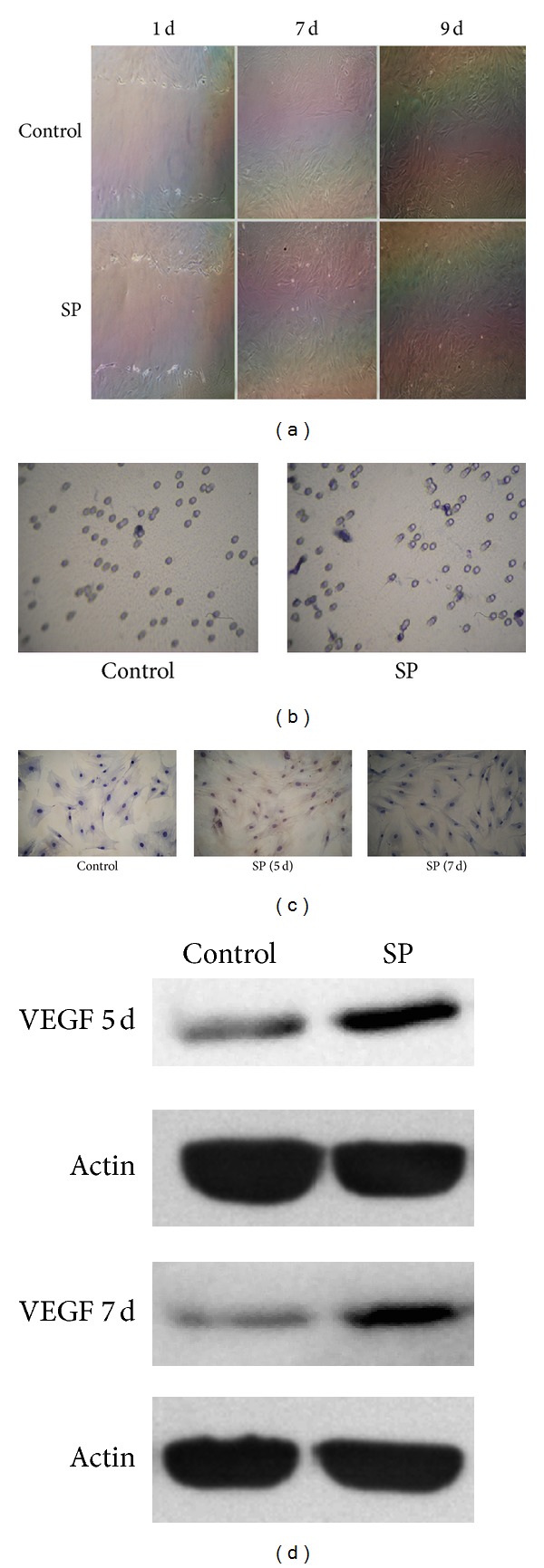
SP increased the cell migration and VEGF expression of BMSCs. SP clearly increased the migration ability of BMSCs as of day 7 and day 9 (a); VEGF was also elevated by SP treatment on day 7 and day 9 ((c), (d)). The transwell migration assays confirmed that the rate of cell migration in the SP group was higher (4.78 ± 1.77) than in the control group (1.11 ± 0.49, (b)).

The scratch recovery assay illustrated that the total distance and speed of the BMSCs were significantly increased by 10^−8^ mol/L SP (Figure 4(a)), especially at 7 days and 9 days after treatment, which was consistent with the VEGF expression results. After SP was applied, the transwell migration assays also demonstrated increased numbers of migration cells (4.78 ± 1.77 in the 10^−8^ mol/L SP group compared with 1.11 ± 0.49 in the control group, Figure 4(b)). The VEGF staining and western blot analysis showed the increased expression of VEGF due to SP (0.2563 ± 0.0095 compared with 0.049 ± 0.004 on 5 days; 0.0347 ± 0.0035 compared with 0 ± 0 on 7 days, Figures 4(c) and 4(d)). The experimental results revealed that after SP treatment, the number of VEGF-positive cells was increased at 7 days and 9 days after treatment, which corresponds to the trend of protein VEGF expression.

## 4. Discussion

Tissue-engineered bone has become a new potential clinical alternative to conventional bone grafts [36]. Though significant advances have been made, a challenge to classical bone tissue engineering strategies has been the lack of vascularization [12]. The implantation of vascular bundles or sensory nerves has been studied as a strategy to overcome this problem. Besides, previous works have shown that the implantation of sensory nerves could promote bone metabolism via neuropeptide action [7–9]. In this experiment, we found that neuropeptide SP could promote osteoblastic differentiation via Wnt signaling and could improve the angiogenic capacity of BMSCs, suggesting that the application of vascular and neurotized bone tissue engineering is theoretically feasible.

SP was observed to stimulate the expression of BMP-2, which is widely used in bone TE construction [12–14]. The experimental results revealed that the SP group and control group showed differences in the expression of BMP-2, indicating that BMP-2 involvement in the bone regeneration process is promoted by SP treatment. The increased BMP-2-positive cells observed in the SP group matched the trend of BMP-2 expression. The local activating effect generated by SP was sustained from 5 days to 7 days during BMSC differentiation. The Wnt/*β*-catenin signaling pathway was suggested to be an upstream activator of BMP2 expression in osteoblasts [37], indicating that the regulating effects exerted by SP on BMP2 may be related to the Wnt signaling activated by SP. The promotion of bone regeneration by SP may be related to the rise of the local BMP-2 expression level in vitro.

Consistent with the present studies demonstrating the osteogenic effect exerted by SP on BMSCs [21, 23, 38, 39], this effect was observed to be related to the activation by SP of the Wnt/*β*-catenin signaling response in this study. Osteoblastic differentiation in vitro is directed by Runx2, the master transcription factor regulating bone formation, and BMSCs can differentiate towards the osteoblastic lineage, which is accompanied by the production of type I collagen and osteocalcin and increased alkaline phosphatase activity [16]. When SP was combined with NK1 receptor antagonist or DKK1, the differentiating effect on the BMSCs was inhibited, suggesting that SP binds to NK1 receptors and activates the Wnt/*β*-catenin signaling pathway in BMSCs. The NK1 receptor that SP acted on in BMSCs and the proliferation mediated by it was revealed and confirmed previously. Consistent with the concentration dependent manner that SP exerted, low concentrations (10^−12^ M) of SP stimulated alkaline phosphatase and osteocalcin expression and upregulated Runx2 protein levels; our results also reflect the phenomena. However, the direct effect on osteoblastic differentiation of SP could be illustrated by ALP staining and mineralization or some detection, which needs further studies in the future.

The activation of the Wnt pathway can be briefly summarized as *β*-catenin accumulation in the cytoplasm, while translocation into the nucleus is also vital; the subsequent transcription of Wnt-related genes including C-myc then begins [40]. We found that SP significantly increased the expressions of the genes and proteins in the Wnt pathway as well as the nuclear translocation of *β*-catenin. NK1 receptor antagonist or DKK1 could inhibit these effects of SP. The translocation of *β*-catenin was observable after 15 min and obvious after 30 min. GSK-3*β* worked as critical regulator of *β*-catenin. P-GSK-3*β* increased due to SP, suggesting that the decreased negative effect of GSK-3*β* was influenced by SP. However, we found that the *β*-catenin mRNAs were unaffected in all groups. Thus, we speculate that the accumulative process of *β*-catenin is not primarily mediated by *β*-catenin mRNA but instead by the state of *β*-catenin, such as phosphorylation or dephosphorylation. The role of Tcf7/Lef1 as either a repressor or activator in Wnt/*β*-catenin signaling is controversial [41, 42]. We found that the treatment of BMSCs with SP led to an increased expression of Lef1 and Tcf7, which appeared to exert a positive effect on the activation of SP-induced Wnt/*β*-catenin signaling.

The migration of BMSCs to the lesion formed blood vessels that supplied oxygen and nutrients, a necessary role in bone repair [31, 32]. VEGF always acted as the primary chemokine in inducing the formation of angiogenesis [43]. We found that SP increased the migration and VEGF expression abilities of BMSCs, which would indicate increased angiogenic capacity. VEGF was always viewed as the primary factor in inducing BMSC migration; the active Wnt signaling contributed to the migration [44], which could partly explain the effect of SP. Considering the contribution of blood supply to bone formation, the angiogenic capacity of BMSCs was leveled up by SP and thus could promote the angiogenesis and then improve osteoblastic differentiation of BMSCs. However, the direct evidence of vessels forming and whether the effect of SP on angiogenesis was mediated by Wnt signaling were not revealed in this study. We have detected the change of VEGF level and active Wnt pathway by SP treatment and it seemed that perhaps the increased formation of new blood vessel was reasonable. Thus, further studies should focus on effect of SP on the vessels formation and more evidence of the related Wnt signaling.

In conclusion, the present study demonstrates that the canonical Wnt signaling may contribute to SP stimulation of the osteogenic differentiation of BMSCs; SP also improved VEGF expression and migration ability. Further in vivo experiments will elucidate the effect of SP on bone tissue engineering construction as well as the relationship between SP and Wnt/*β*-catenin signaling, thus furthering our understanding of the role of vessels and nerves in bone tissue engineering.

## Figures and Tables

**Table 1:** 

Gene	Sequence		Predicted length (bp)
NK1	F:	GGCCTTCGACAGATACATGG	140
	R:	TCTCTGTGGTGGAGTAGTAG	

ALP	F:	CCTTGAAAAATGCCCTGAAA	191
	R:	CTTGGAGAGAGCCACAAAGG	

Osteocalcin	F:	CATGAGGACCCTCTCTCTGC	153
	R:	AGGTAGCGCCGGAGTCTATT	

Col1a1	F:	TGGTCCTCAAGGTTTCCAAG	123
	R:	TTACCAGCTTCCCCATCATC	

Runx2	F:	GAGCTACGAAATGCCTCTGC	173
	R:	GGACCGTCCACTGTCACTTT	

CCND1	F:	GCGTACCCTGACACCAATCT	180
	R:	CTCTTCGCACTTCTGCTCCT	

c-myc	F:	GCTCCTCGCGTTATTTGAAG	152
	R:	TTCTCTTCCTCGTCGCAGAT	

*β*-Catenin	F:	CTCCCCTGACAGAGTTGCTC	187
	R:	ATGTCCAGTCCGAGATCAGC	

Tcf7	F:	GCACGGGATAACTACGGAAA	99
	R:	AAAGCGAGCACGACATTTCT	

Lef1	F:	TAACAAGGGCCCCTCCTACT	198
	R:	CCTGGAGAAAAGTGCTCGTC	
